# Genome sequence alterations detected upon passage of *Burkholderia mallei *ATCC 23344 in culture and in mammalian hosts

**DOI:** 10.1186/1471-2164-7-228

**Published:** 2006-09-05

**Authors:** Claudia M Romero, David DeShazer, Tamara Feldblyum, Jacques Ravel, Donald Woods, H Stanley Kim, Yan Yu, Catherine M Ronning, William C Nierman

**Affiliations:** 1 The Institute for Genomic Research, 9712 Medical Center Drive, Rockville, MD 20850, USA; 2 Bacteriology Division, United States Army Medical Research Institute of Infectious Diseases, Fort Detrick, MD 21702, USA; 3 Department of Microbiology and Infectious Diseases, University of Calgary Health Sciences Centre, Calgary, Alberta T2N 4N1, Canada; 4 The George Washington University School of Medicine, Departmentof Biochemistry and Molecular Biology, 2300 Eye Street NW, Washington, DC 20037, USA

## Abstract

**Background:**

More than 12,000 simple sequence repeats (SSRs) have been identified in the genome of *Burkholderia mallei *ATCC 23344. As a demonstrated mechanism of phase variation in other pathogenic bacteria, these may function as mutable loci leading to altered protein expression or structure variation. To determine if such alterations are occurring in vivo, the genomes of various single-colony passaged *B. mallei *ATCC 23344 isolates, one from each source, were sequenced from culture, a mouse, a horse, and two isolates from a single human patient, and the sequence compared to the published *B. mallei *ATCC 23344 genome sequence.

**Results:**

Forty-nine insertions and deletions (indels) were detected at SSRs in the five passaged strains, a majority of which (67.3%) were located within noncoding areas, suggesting that such regions are more tolerant of sequence alterations. Expression profiling of the two human passaged isolates compared to the strain before passage revealed alterations in the mRNA levels of multiple genes when grown in culture.

**Conclusion:**

These data support the notion that genome variability upon passage is a feature of *B. mallei *ATCC23344, and that within a host *B. mallei *generates a diverse population of clones that accumulate genome sequence variation at SSR and other loci.

## Background

*Burkholderia mallei *is a nonmotile, Gram-negative bacillus and the causative agent of a severe disease known as glanders. Humans are accidental hosts of *B. mallei*; the natural hosts for *B. mallei *are horses, donkeys and mules [1-3]. There are two distinctive forms of glanders, the acute form characterized by septicemia and pulmonary infection and the chronic form characterized by suppurative infection [4].

The complete genome sequence of *B. mallei *ATCC 23344, a highly pathogenic clinical isolate [5,6], has been recently published [7]. The genome of *B. mallei *ATCC 23344 contains more than 12,000 simple sequence repeats (SSRs) within coding areas and in putative promoter regions. It also contains numerous insertion sequence elements. SSRs are repetitive DNA made of identical or mixed repeat units. SSRs have been known to be highly polymorphic and to be distributed throughout the genomes of eukaryotes [8,9]. The presence of prokaryotic SSRs is well documented [10-14]. Studies using *Saccharomyces cerevisiae *and *Escherichia coli *as model organisms have shown that the variability in these repeats may be due to slipped-strand mispairing (SSM) during DNA replication [15] resulting in insertions or deletions (indels) of repeat monomeric units [12,16]. These indel mutations may destabilize an essential regulatory structure or hamper gene function or, if located within coding regions of the gene, may cause frameshifts in the coding reading frame or otherwise alter the amino acid sequence of the protein product of the gene. SSRs have been used as markers for the identification of pathogenic bacteria and have been implicated as an important prerequisite for bacterial phase variation and adaptation [17-19].

Observations on glanders immunity make the presence of such high levels of SSRs in the *B. mallei *genome particularly intriguing. Immunity to glanders is not conferred by a prior infection [4,23]. At present, there are no vaccines that induce protective immunity in the horse or sterilizing immunity in mice [6]. Serum from a glanderous horse does not confer immunity on a recipient horse, and pathogenic strains have been reported to lose virulence on laboratory passage and to regain it upon subsequent animal passage [4]. A mechanism of reversible genome alteration mediated possibly through SSRs mutations or insertion sequence elements on passage could account for all of these observations.

To the best of our knowledge, no studies reporting genome sequence changes during short term acute infections have been reported for any bacterial pathogen. In many human infections such as HIV, tuberculosis, leprosy, and malaria, hosts and pathogens coexist for years or decades. With the exception of HIV/AIDS, little is know about the adaptation of the pathogen through genome alterations during these chronic infection periods. Genome sequence alterations have been explored in *Pseudomonas aeruginosa *in an opportunistic infection of a single human cystic fibrosis patient by genome sequence analysis of two single colony isolates at two times 8 years apart [24]. Over this period 68 genome sequence alterations were detected, 49 SNPS and 19 insertions/deletions. Most insertions/deletions were 1 to 3 bases with no SSR association noted.

Since *B. mallei *has been used previously as a biological weapon [25,26], with potential for future use by terrorists, studies on its mechanisms of pathogenesis and immunity are of great importance. In this report, we explore the issue of genome stability upon passage of *B. mallei *in culture and in several mammalian hosts, including human. We report that an unprecedented level of bacterial genome alteration occurs in *B. mallei *upon short term passage. While RNA viruses incur consequential rapid genome variation as a major component of their strategy for escaping the host immune response, the level of genome variation reported here on *B. mallei *passage represents the first report of such variation for a bacterial pathogen.

## Results

### SSRs within the *B. mallei *ATCC 23344 genome

The distribution of the 12,547 SSRs within the *B. mallei *genome from an overview perspective appears to be random: 2,997 (23.9%) are intergenic and 9,550 (76.1%) are located within the coding regions of genes (Table 1). This approximates the allocation of genomic DNA to the intergenic (14.4%) and coding fractions of the genome (85.6%). In addition, when evaluating genes by functional category, the distribution of genes containing SSRs in each category reflects that in the genome. Heteropolymer repeats (11,041) are more abundant than homopolymer repeats (1,506). SSRs consisted of up to 111 tandem copies of the repeat unit, which were found to be as long as 14 nucleotides. The base composition of the SSR repeat units is consistent with the base composition for the overall genome, 60 to 68% GC.

### Indels within intergenic regions

After passage, a total of 33 indels were found within non-coding or intergenic regions relative to the reference genome sequence of *B. mallei *ATCC 23344: nine in the laboratory culture passaged isolate, eight in the mouse spleen isolate, eight in the horse lung isolate, three in the human liver isolate, and five in the human blood isolate (Table 2). Twenty-four indels were located within SSRs, ten indels were near or within a promoter sequence, and twelve contained palindromic sequences. Such palindromic structures have been shown to perform many important biological roles including termination of transcription. All indels identified, except for indels 6 in the mouse spleen isolate and 8 in the lab culture and indels 3 in the human liver isolate and 3 in the human blood isolate, were different.

#### Intergenic indels within SSRs

Among the intergenic indels, those located within SSRs are most common (24/33). The repetitive sequence units differed from 7 to 14 nucleotides and each unit was repeated from three to 111 times (Table 2). Eight of these indels within SSRs were located close to promoter areas and six were close to palindromic sequences.

#### Intergenic indels not within SSRs

A total of nine intergenic indels not located within repetitive units were found (Table 2). All nine were near promoter or palindromic regions.

### Indels within coding regions

Sixteen indels were found within coding regions of the passaged isolates; four indels in the lab passaged isolate, two indels in the mouse spleen isolate, three indels in the horse lung isolate, four indels in the human liver isolate, and three indels in the human blood isolate (Table 3). Only seven indels are within SSRs, and 14 out of the 16 indels created a frameshift mutation within the encoded protein. All indels identified except for two pairs, indels 1 and 2 from human blood and liver, and indel 3 from human liver and blood, were different, suggesting that there are numerous sites of elevated mutation in the *B. mallei *genome that can potentially be altered in some individuals in the bacterial population upon passage.

#### Coding region indels within SSRs

Only seven indels within repetitive sequence units differing from six to 12 nucleotides were found within coding regions (Table 3). SSRs with a monomer length that is not multiple of three and located within gene coding regions can significantly alter the coding potential of a given transcript. Five of the seven indels within SSRs with unit repeat of seven and eight nucleotides caused frameshift mutations resulting in altered amino acids from the point of mutation and premature truncation likely producing an altered or non-functional protein. These five affected proteins are annotated as either hypothetical or conserved domain proteins. The other two SSR-containing indels with unit repeat of six and 12 nucleotides only add two or remove four amino acids from the encoded protein. One of these proteins encodes a penicillin-binding protein, PBP-1c, which normally functions in cell wall synthesis and beta-lactam resistance.

#### Coding region indels not within SSRs

Most indels in coding regions (nine of 16) were not located within SSRs (Table 3). These indels result from uncorrected replication errors possibly reflecting a lower level of DNA repair activity relative to other bacteria (see Discussion).

### Do in vivo accumulated indels alter gene expression patterns?

In order to determine if the genome sequence alterations that accumulated during mammalian passage altered the expression of the genes at the site of the indels, expression profiling of the two human isolates (FMH and JHU) of *B. mallei *was accomplished relative to the unpassaged parental strain (i.e. ATCC 23344) after growth in culture using the whole genome glass slide amplicon array and protocols previously described [7]. When the FMH and JHU samples were each hybridized against the ATCC 23344 references, only a very limited number of genes showed altered expression ratios of over 2 fold. For the FMH isolate only 59 genes were at a 2 fold or higher level more while only two were at a 2 fold or more lower level (Table 4). For JHU the respective numbers were 17 and 3 (Table 5) with 13 of the up-regulated genes in common between the two strains. Two of the > 2X up-regulated genes were located very close to genes of the mutant site (Table 6A). Genes co-located with the indel mutations in some cases did show expression ratio alterations (Table 6B). To assess the integrity of this data set, additional preparations of RNA from the unpassaged ATCC 23344 strain were grown and the RNA isolated on separate days. These two RNAs were hybridized against each other. The results of this hybridization showed no gene to be 2 fold up regulated in either preparation relative to the other. In this experiment approximately half (3156) of the genes showed RNA level within 93% of each other (log_2 _of 0.10). In contrast for the FMH vs. ATCC 23344 experiment only 1767 genes were the same within this range and for the JHU vs. ATCC 23344 only 1634 genes were within this range. These data suggest that the transcription profiles of the JHU and FMH isolates when grown in culture are similar but modestly distinct relative to each other and relative to the unpassaged ATCC 23344 strain.

## Discussion

We have detected what appears to be a high level of genome instability in *B. mallei *upon passage in culture or in animals. Much of this instability is through alteration in the number of repeat units within SSRs. If indeed these SSRs function as sites for elevated levels of mutation on passage, this affords tremendous potential for genome variation within an animal host. With this potential in mind, we sequenced *B. mallei *ATCC 23344 to various levels of coverage after passage in culture and in mouse, horse, and two isolates from an accidental infection of a biodefense scientist [5].

We observed indel mutations both at SSR sites and other locations with few or no SNPs resulting upon passage. In *Escherichia coli *an increase in the rate of mutation under stress conditions has been documented (reviewed in [27]). The mutations are manifest as amplifications and point mutations [28]. These mutations are mediated by an error-prone DNA polymerase, DNA polymerase IV, which is regulated by RpoS, the stress response sigma factor [29], the heat-shock chaperone GroES [29], and polyphosphate kinase [30]. The *B. mallei *indels observed upon passage could be the consequence of such a stress-induced enhanced mutation rate upon host immune response stress or upon that stress leading to reduced growth rate upon entering stationary phase in culture. That this may be true is suggested by the observation that the *B. mallei *genome contains homologs of the *E. coli *proteins demonstrated to participate in this process.

The mutations reported here upon passage of *B. mallei *are indels at SSRs and other sites. Indels at SSRs that change the number of repeat units are the result of slip-strand mispairing during replication [15]; reviewed in [31]. Elevated SSM rates at SSRs may be caused by an increased likelihood of both slippage and misalignment [32,33]. Such replication errors are repaired by the mismatch repair activities of the *mutS*, *mutL*, and *mutM *gene products. Indels in particular are a hallmark of reduced mismatch repair. Although *B. mallei *does possess homologs to these mut genes, the role of these repair genes in the generation of indels upon passage remains to be elucidated.

The findings in this study also suggest that the genomic distribution of SSR-associated indels is nonrandom across coding and noncoding regions. SSR associated indels constitute a large fraction of noncoding DNA indels and are relatively rare in protein-coding regions. These SSRs located in intergenic regions may affect gene transcription and activity. It has been previously shown that important regulatory sequence elements in viruses are often duplicated within promoters, either directly repeated, or as inverted copies of sequence segments [34]. Studies conducted with geminivirus and nanovirus families of DNA plant viruses revealed that DNA elements including those containing small internal palindromic sequences play a significant role in the enhancement of transcription and contribute to regulation of *in vivo *viral gene expression during plant infection [35]. It would not be surprising if *B. mallei *uses a similar mechanism for regulation of gene expression during *in vivo *infection.

Non-SSR associated indels in these passaged isolates reflect the possible presence of reduced levels of replication associated DNA repair resulting in a large number of indels on passage of *B. mallei*. This process of genome alteration on passage is likely distinct from that leading to SSR associated alterations.

The evolutionary history of *B. mallei *may be contributing to its ability to tolerate this level of genome instability. The *B. mallei *genome structure [7] demonstrates that *B. mallei *is a reduced and rearranged version of *B. pseudomallei *that has evolved from a versatile pathogenic soil organism to an obligate mammalian parasite. This process of reduction and rearrangement has been mediated through the numerous IS elements present in the *B. mallei *genome and has left multiple intact genes that are no longer necessary to its life as a mammalian parasite. As an example, it possesses a large set of mostly intact, relative to *B. pseudomallei*, chemotaxis and motility genes while it is non-flagellated and non-motile. Such genes may provide a target for genome alterations, such as gene decay, that would be under no selection.

In general, genome variation as an infection progresses is a common strategy for pathogenesis employed by RNA viruses to escape clearing by the host immune system. Such large scale genome instability is not known to be a regular feature of pathogenic bacteria. To the best of our knowledge, no systematic study has been reported on the stability of bacterial genomes upon passage in a mammalian host during a short term acute infection. In *Bacillus anthracis*, geographically distinct isolates differ in genome sequence by only few SNPs [36], suggesting that the *B. anthracis *genome would prove to be very stable upon passage. In contrast to *B. mallei*, there is little genome sequence variation among the *B. anthracis *isolates (reviewed in [37]). On a whole genome scale, much of the increased rate of indels accumulated in *B. mallei *ATCC 23344 upon passage may simply be due to the large number of these mutable SSR sites within the *B. mallei *genome. The estimated rate of unrepaired DNA replication errors leading to SNPs in *B. anthracis *is approximately 10^-10 ^changes per nucleotide per generation [38]. In *B. mallei*, the rate of SNPs generated upon passage in the human and the horse was observed to be very low.

For other bacteria, including *B. mallei*, the genome diversity within the species includes major insertions and deletions, eliminating the possibility of inferring anything about genome stability upon passage based on the species genome diversity. One of the SSR-containing indels that we report here encodes penicillin-binding protein (PBP-1c) that is usually involved in cell wall synthesis and beta-lactam resistance. A study done by Jones *et al*. [39], reported a novel function for a PBP-1a in group B streptococci. This study showed that this protein *in vivo *promoted resistance to phagocytic killing independent of capsular polysaccharide. It might be possible that within the mouse the lack of one repeat unit and further loss of four amino acids leads to a conformational change in this membrane protein that allows for a novel function or altered host immune response in vivo. If true, this could be a mechanism used by *B. mallei *for evasion of immune recognition and clearance in vivo.

One potential SNP was identified in the human blood isolate. This SNP, a C-G substitution, occurred in gene BMAA0914, annotated as choline dehydrogenase. However, since we did not resequence the SNP its validity is unconfirmed. We conclude from the SNP analysis that, in contrast to our observation on the accumulation of indels, SNPs are not generated to any consequential extent upon passage. SNP analysis was not performed on the culture and mouse isolates due to the 4X sequence coverage of these isolates. This is sufficient coverage for indel analysis because indels involve multiple base positions, and the sequence quality across the region of the indels can be used to ascertain the validity of the detected indels. Validating single base calls in a sequence requires more coverage so that SNP analysis was performed only for the two human isolates sequenced to 9X coverage. Further studies may include increasing the sequence coverage of the culture and mouse isolates in order to evaluate the SNPs in these genomes.

The high sequence coverage of the horse passaged isolate and of the two human isolates allows a calculation of the level of genome variation upon passage in these hosts. The altered bases in each instance, 53 of 5.8 Mb for the horse isolate, 60 of 5.8 Mb for the human blood isolate, and 42 of 5.8 Mb for the human liver isolate gives an average level of genome sequence alteration upon passage of 8.9 e^-6^. While this is less than what would be observed upon passage of HIV, we postulate that it is at the very high end of what would be observed upon passage of other pathogenic bacteria. We further postulate that this genome instability is a design feature of the structure and replication machinery of the bacterium and is an integral component of the organism's approach to survival within the mammalian host.

The two isolates from the single human patient further afford the opportunity to explore the *B. mallei *population structure once it takes up residence in the mammalian host and the level of sequential events of genome alteration upon passage in the human host. The presence of multiple indels, only two of which are common to the two isolates, suggests that the organism is maintained not as a clonal population once in the host but as a population of variant individuals.

*B. mallei *genome sequence alterations accumulated and fixed during the course of an infection in a mammalian or human host would not be expected to reduce the fitness of the individual bacterium within the host. If fitness of an individual were reduced, it is expected that the individual would be lost from the population. Thus, most alterations of genome sequence that accumulate within a host would be expected to have a minimum adverse consequence for bacterial expression patterns within the host, while infrequently increasing fitness of the mutant individual. Indeed, we have observed that those genes that are orthologous between *B. mallei *and *B. pseudomallei *were expressed largely at identical levels within a mouse host (Kim et al. unpublished), suggesting that expression patterns within a host are well conserved in these *Burkholderia *pathogens. The human isolates studied here when grown in culture might be expected to exhibit some alteration in gene expression pattern after the accumulation of alterations in the host. Indeed, a modest number of genes exhibit modest alterations in levels of expression with several of these genes near the sites of the indel mutations (Table 4). All of the indels detected within coding regions in the FMH and JHU isolates cause frameshifts, four in JHU and three in FMH. These frameshifts, especially in some of the regulatory genes, may account for the altered *in vitro *patterns of expression reported here.

## Conclusion

The inability of a mammalian host to gain immunity to glanders infection, as well as its past and potential use as a biological weapon, make understanding *B. mallei *pathogenicity, virulence, and mechanisms for evading the host immune response of critical importance to the modern world. We report here the occurrence of genome variation in *B. mallei *ATCC 23344 upon its passage through several mammalian hosts at a level unprecedented in bacteria. We also report that two strains isolated from the infection of a single human host exhibit distinct altered gene expression patterns relative to the unpassaged strain when grown in culture. This genome instability upon passage may have implications for vaccine development and treatment of this very serious disease.

## Methods

### Bacterial isolates and DNA preparation

#### Laboratory passage

A glycerol stock of *B. mallei *ATCC 23344 was used to inoculate a petri plate containing Lennox LB agar (Sigma) with 4% glycerol (LBG). The plate was incubated at 37°C for 2 days and an inoculating loop was used to transfer cells from the primary quadrant to a new LBG plate. The remainder of the primary quadrant was harvested with a sterile cotton swab, resuspended in LBG broth, mixed with an equal volume of 40% glycerol, designated "laboratory passage #1", and stored at -70°C. This process was repeated, without interruption, a total of 23 times. Ten microliters of "laboratory passage #23" was used to inoculate a LBG plate and isolated colonies were randomly chosen after growth at 37°C for 2 days. One of the colonies designated SLP 1 was grown in 3 ml of LBG broth overnight at 37°C and genomic DNA was prepared following a previously described protocol [40]. SLP1 was selected for subsequent sequencing.

#### Mouse passage

BALB/c mice were aerogenically infected with approximately 1 LD_50 _(1,000 cfu) of *B. mallei *ATCC 23344. An infected mouse was sacrificed thirty-three days post-challenge and the spleen was removed, homogenized, serially diluted in 0.85% NaCl, and cultured on LBG plates for 2–3 days at 37°C. The spleen contained > 10^7 ^cfu/g, demonstrating that the animal was acutely infected with *B. mallei*. Isolated colonies were randomly selected and grown in 3 ml of LBG broth overnight at 37°C and genomic DNA was prepared from each culture [40]. One designated CMI1 was selected for subsequent sequencing.

#### Horse passage

A single colony isolate of *B. mallei *was obtained from a single horse from an experiment involving six horses used in a study to characterize glanders disease progression [41]. Animals were housed in biosafety level 3 containment at the National Centre for Foreign Animal Disease in Winnipeg, Manitoba, where all experiments were performed. Prior to the beginning of experimentation, animals were allowed to acclimatize to their surroundings for a 2-week period. Horses were anesthetized and inoculated intratracheally with 4 mL of a suspension containing 1 × 10^10 ^*B. mallei *ATCC 23344 cfu/mL [41]. Seven days following inoculation, horses were sacrificed, and lung samples were taken for *B. mallei *isolation. Approximately 5 g of tissue were placed in 3 ml PBS in conical tubes. The tissues were homogenized with a Brinkman Polytron Homogenizer. Homogenates in PBS were plated on four different media including BHI agar (Difco) containing 5% sheep blood and 4% glycerol, Columbia CAN Agar (Difco) containing 5% sheep blood, a selective trypticase soy-based agar containing 1% glycerol, 1000 units polymyxin E, 1250 units bacitracin and 0.25 mg actidione per 100 ml [11], and MacConkey Agar (Difco). A single colony isolate designated GB8 horse 4 was selected for sequencing. Genomic DNA was prepared following a previously described protocol [40].

#### Isolates from a laboratory acquired infection

Two isolates were obtained from laboratory acquired infection [5]. These *B. mallei *ATCC 23344 human isolates were obtained from liver, designated JHU, and from blood, designated FMH, approximately 2 months after initial infection, and genomic DNA was prepared from each culture [40].

All animals used in this research project were cared for and used humanely according to the following policies: The U.S. Public Health Service Policy on Humane Care and Use of Animals (1996); the Guide for the Care and Use of Laboratory Animals (1996); and the U.S. Government Principles for Utilization and Care of Vertebrate Animals Used in Testing, Research, and Training (1985). All NCI-Frederick animal facilities and the animal program are accredited by the Association for Assessment and Accreditation of Laboratory Animal Care International.

### Shotgun sequencing and assembly

Shotgun sequencing was performed as described [7]. Sequence was accumulated to achieve 4X genome coverage for the culture and mouse isolates. Sequence was accumulated to achieve 8X genome coverage of the horse isolate and 9X coverage of the two human isolates. These genomes were assembled using the AMOScmp assembler [42] with the *B. mallei *ATCC23344 genome sequence as the assembly reference genome. This assembler uses a very closely related genome sequence as a reference that is used to guide the assembly of the shotgun sequence reads into contigs.

### Identification of SSRs and SNPs

A bioinformatics pipeline was developed consisting of custom scripts that identify SNPs and indels when a shotgun genome assembly is compared to a closed reference genome (*B. mallei *ATCC 23344). The scripts integrate the whole genome alignment tool, MUMmer [43] to map each contig to the reference genome sequence and identify polymorphic sites. For each match, SNPs and indels are extracted and automatically validated based on sequence coverage and quality values of the region where the polymorphism is detected. Briefly, a SNP is considered of high quality when its underlying sequence comprised at least three sequencing reads with an average Phred score [44,45] greater or equal to 30 on both the reference and the query genome. Each sequence difference was further reviewed and scored manually. When the indel report was inconclusive, the underlying sequence traces and the consensus sequence were analyzed using Cloe, the TIGR sequence editor program, to correct scoring of the indel. SNPs were identified and validated only for the two human isolates since they were sequenced to high coverage. Indels were identified and validated for all of the isolates.

### Expression analysis

A whole genome PCR amplicon DNA microarray for B. mallei were fabricated as previously described [7]. Total RNA was isolated from in vitro cultures in LBG medium of B. mallei ATCC 23344, FMH, and JHU. The OD_600 _of the samples at harvest were all 0.55. The RNAs from FMH and JHU were labeled and hybridized to the array using the ATCC 23344 RNA as the reference using protocols as described. Flip-dye replicates were performed for all analyses. Two B. mallei ATCC 23344 samples grown to an O.D._600 _of 1.0 on separate days and total RNA was extracted. These RNA samples were hybridized against each other as a control for the JHU vs. ATCC 23344 hybridization and the FMH vs. ATCC 23344 hybridization.

### Microarray data availability

Microarray expression data presented in this manuscript are available through ArrayExpress [Array Express at EBI: [] with accession numbers A-MEXP-206 (array design) and E-MEXP-/// (experimental data).

## Abbreviations

SSR – Simple Sequence Repeat

indel – Insertion or Deletion

SNP – Single-Nucleotide Polymorphism

## Authors' contributions

DD passaged strains in culture and mouse and hosted human isolates, prepared genomic DNA from these strains and isolates, and designed experiments and prepared RNA for microarray expression profiling. CM Romero analyzed indels and SNPs, and prepared the initial manuscript draft. WCN designed the study and analyzed the microarray data, drafted sections of text, and performed editorial review. JR wrote and ran software for analyzing indels and SNPs in the Burkholderia isolates. CM Ronning performed informatic analysis and validation, drafted text sections, and edited and organized the manuscript for submission. DW conducted horse experiments, cultures, and genomic DNA, and reviewed the manuscript. HSK designed and supervised microarray hybridization experiments and performed initial microarray data analysis. YY performed microarray experiments and data analysis. TF directed the sequencing and sequence editing of the strains, and validated SNP/indel data. All authors read and approved the final manuscript.

## Figures and Tables

**Table 1:** Perfect simple sequence repeats (SSRs) identified in the *B. mallei *ATCC 23344 genome.

Chromosome	Coding			Intergenic	Total
			
	5' end	Middle	3' end		
1	1809	1811	1786	1789	7195
2	1401	1433	1310	1208	5352

Total	3210	3244	3096	2997	12547

Locations of the SSRs in the genome are denoted with the coordinates of their start and end points (i.e. match 5' end and 3' end) in the relevant chromosomes (i.e. 1 or 2) and also with their relative positions within the coding region of a gene: 5' end, middle, and 3' end.

**Table 2:** Indels within intergenic regions.

		SSR					Nearest ORF	
					
Isolate	Indel	Unit	Reference	Query	Near Promoter	Near Palindrome	Locus	Annotation
Lab Culture	1	TTGCCCGGCGA	7	6	yes	no	BMA1136	Hypothetical protein
	2	TTCGACGC	29	28	yes	no	BMA2062	Hypothetical protein
	3	AGTCGGCA	38	39	no	no	BMA1136	Hypothetical protein
	4	CGATTGCCCGG	7	8	yes	no	BMA1138	ABC transporter, putative
	5	GGGGCTTC	9	8	no	no	BMA2063	Transcriptional regulator
	6	TGCGCGA	19	15	no	no	BMA2374	THUMP domain protein
	7	No (-G)	NA	NA	yes	no	BMAA0389	Hypothetical protein
	8	CTGTCGTG	21	22	no	no	BMAA0376	Transporter
	9	GTGCGAT	19	20	no	no	BMAA1878	Transcriptional regulator
								
Mouse Spleen	1	GAGGCGT	26	25	no	no	BMA2774	Secretory path protein
	2	No (+TT)	NA	NA	yes	no	BMA1596	Acetyltransferase
	3	CGCGAGG	23	22	yes	no	BMAA0247	Oxidoreductase
	4	GTGGCGA	7	6	no	no	BMAA0375	Transcriptional regulator
	5	AAGTTCCG	3	4	yes	no	BMAA0242	Acyl-CoA dehydrogenase
	6	CTGTCGTG	21	22	no	no	BMAA0376	Transporter
	7	TGGCGTT	26	27	yes	no	BMAA0242	Acyl-CoA dehydrogenase
	8	GAAAGAGAC	10	11	yes	no	BMAA0815	DNA-binding regulator
								
Horse Lung	1	GTGAGCC	13	14	no	no	BMA0984	Hypothetical protein
	2	No (-C)	NA	NA	no	yes	BMAA1128	ABC Transporter
	3	GGGAAACGCGAAAC	6	5	yes	no	BMAA1873	Hypothetical protein
	4	No (-C)	NA	NA	no	yes	BMAA1868	Aconitate hydratase
	5	No (+C)	NA	NA	no	yes	BMAA1420	Synthetase protein
	6	No (+T)	NA	NA	no	yes	BMAA1237	Carboxyvinyltransferase
	7	GCGAAAC	5	6	no	yes	BMAA1872	Chemotaxis protein
	8	GATGAGC	19	20	no	yes	BMAA0612	Signal sequence protein
								
Human Liver	1	GGCAAGTC	38	40	no	yes	BMA1135	Drug resistance transporter
	2	No (-C)	NA	NA	no	yes	BMAA1868	Aconitate hydratase
	3	GTGCTGTC	21	22	no	yes	BMAA0375	Transcriptional regulator
								
Human Blood	1	TTGGCGC	111	109	no	no	BMAA1866	Conserved hypothetical protein
	2	AAGCAGC	42	40	no	yes	BMAA0117	6-phosphofructokinase (pfk)
	3	GTGCTGTC	21	22	no	yes	BMAA0375	Transcriptional regulator
	4	No (-C)	NA	NA	no	yes	BMAA1128	ABC transporter
	5	No (-C)	NA	NA	no	yes	BMAA1868	Aconitate hydratase

NA: Not applicable.

**Table 3:** Indels within coding regions.

Isolate	Indel	Reference Protein Length	Frameshift Length (bp change)	Query Protein Length	Locus	Annotation
Lab Culture	1	638 aa	29 aa (+C)	No stop codon^b^	BMAA1927	Hypothetical Protein
	2	154 aa	50 aa (-T)	75 aa	BMA1435	Hypothetical Protein
	3	145 aa	64 aa (-G)	85 aa	BMA2147	N utilization substance protein B
	4	711 aa	586 aa (+A)	No stop codon^b^	BMA2914	Oxidoreductase
						
Mouse spleen	1^a^	942 aa	No	938 aa	BMAA0680	Penicillin-binding protein
	2	357 aa	13 aa (+G)	321 aa	BMA0161	Rod shape-determining protein MreC
						
Horse Lung	1	787 aa	525 aa (+G)	721 aa	BMAA0367	Acetyltransferase, GNAT family
	2^a^	136 aa	No	138 aa	BMAA0623	Hypothetical protein
	3^a^	120 aa	66 aa	100 aa	BMA2996	Hypothetical protein
						
Human Liver	1^a^	659 aa	52 aa	62 aa	BMAA0729	Hypothetical protein
	2^a^	122 aa	69 aa	85 aa	BMA3028	Conserved domain protein
	3	No translation	49 aa (+A)	361 aa	BMAA1903	Conserved hypoth. protein
	4	685 aa	164 aa (+T)	328 aa	BMA0685	Vit. B12 receptor BtuB, putative
						
Human Blood	1^a^	122 aa	69 aa	85 aa	BMA3028	Conserved domain protein
	2^a^	193 aa	157 aa	No stop codon^b^	BMAA0789	Hypothetical protein
	3	No translation	49 aa (+A)	361 aa	BMA1903	Conserved hypoth. protein

^a^Coding region indels within SSRs. Mouse spleen 1^a^: repeat unit AACACCGAACCG; Horse lung 2^a^: repeat unit GGTGCC, 3^a^: repeat unit GAGCGGT; Human liver 1^a ^: repeat unit CGAGTCAT extra copy in reference, 2^a^: repeat unit GCCGATT extra copy in query; Human blood 1^a^: GCCGATT extra copy in query, 2^a^: GCGCCTC two extra copies in reference.

^b^Reference protein lost the stop codon at the original position due to the frameshift; query protein has a new stop codon in a different position.

**Table 4:** Expression profile of unpassaged reference strain (ATCC 23344) relative to the human blood isolate (FMH), expressed as the log_2 _ratio of intensities.

**Ratio**	**Locus**	**Annotation**
2.15	**BMAA0446**	Rhs element Vgr protein
1.64	BMAA1895	conserved domain protein
1.60	**BMAA0090**	lipoprotein, putative
1.56	**BMAA2014**	hypothetical protein
1.52	BMAA1663	hypothetical protein
1.51	**BMAA0618**	hypothetical protein
1.49	BMAA0810	YadA-like C-terminal region protein
1.44	**BMA2461**	C4-dicarboxylate transport protein
1.43	BMAA2044	conserved hypothetical protein
1.42	**BMA3164**	hypothetical protein
1.42	BMA0632	conserved hypothetical protein
1.42	**BMAA1384**	hypothetical protein
1.41	BMAA1999	hypothetical protein
1.41	BMAA0682	hypothetical protein
1.39	**BMA2875**	hypothetical protein
1.38	**BMAA1865**	conserved hypothetical protein
1.38	BMAA0268	rubrerythrin
1.38	BMA1006	hypothetical protein
1.34	BMA0985	hypothetical protein
1.34	**BMA0017**	hypothetical protein
1.31	BMA0040	conserved hypothetical protein
1.31	**BMAA0922**	drug resistance transporter, EmrB/QacA family
1.30	BMA0813	conserved hypothetical protein
1.29	BMA0833	DNA-binding response regulator
1.28	**BMA2979**	acyltransferase family protein
1.28	BMAA0059	conserved hypothetical protein
1.26	BMAA0976	dipeptide ABC transporter, permease protein, putative
1.25	BMAA2019	hypothetical protein
1.25	BMAA1885	membrane protein, putative
1.24	BMA2676	DNA-binding response regulator
1.24	BMA1631	hypothetical protein
1.20	BMAA0737	Rhs element Vgr protein
1.20	BMA1854	Ser/Thr protein phosphatase family protein
1.19	**BMA0859**	hypothetical protein
1.18	BMA0036	hypothetical protein
1.14	BMAA1974	conserved hypothetical protein
1.13	BMAA0656	hypothetical protein
1.13	BMA1633	dioxygenase, TauD/TfdA
1.12	BMAA1879	hypothetical protein
1.11	BMAA0651	H-NS histone family protein
1.09	BMAA0585	secretory lipase family protein
1.09	BMAA0076	conserved domain protein
1.08	BMAA0178	hypothetical protein
1.08	BMAA0053	membrane protein, putative
1.07	BMAA0935	hypothetical protein
1.06	BMAA0204	ortho-halobenzoate 1,2-dioxygenase beta-ISP protein OhbA
1.06	BMAA1888	hypothetical protein
1.05	BMAA2035	stress response protein
1.05	BMA2983	ethanolamine ammonia-lyase heavy chain
1.05	BMAA1652	MoaC domain protein
1.04	BMA1132	hypothetical protein
1.04	BMAA1916	hypothetical protein
1.04	BMAA0112	hypothetical protein
1.04	BMAA1627	type III secretion inner membrane protein SctS
1.02	BMAA0391	monooxygenase family protein
1.01	BMAA0752	hypothetical protein
1.00	BMA3275	oxidoreductase, GMC family
1.00	BMAA0470	hypothetical protein
1.00	BMAA0061	RNA polymerase sigma-70 factor, ECF subfamily
-1.05	BMAA0866	hypothetical protein
-1.06	BMA1987	dTDP-4-dehydrorhamnose reductase

Genes exhibiting ≥ 2-fold intensity (mRNA abundance) difference are listed. Highlighted genes are also differentially expressed in the human liver isolate (JHU) (see Table 5).

**Table 5:** Expression profile of the unpassaged reference strain (ATCC 23344) relative to the human liver isolate (JHU), expressed as the log_2 _ratio of intensities.

**Ratio**	**Locus**	**Annotation**
1.82	**BMAA0090**	lipoprotein, putative
1.81	BMA3047	heat shock protein, Hsp20 family
1.79	**BMAA0446**	Rhs element Vgr protein
1.68	BMA3048	heat shock protein, Hsp20 family
1.46	**BMA2461**	C4-dicarboxylate transport protein
1.24	BMA0118	RNA polymerase sigma factor RpoD, putative
1.22	**BMAA0922**	drug resistance transporter, EmrB/QacA family
1.19	**BMAA0618**	hypothetical protein
1.18	**BMAA1865**	conserved hypothetical protein
1.16	**BMA2979**	acyltransferase family protein
1.15	**BMA0017**	hypothetical protein
1.14	**BMA3164**	hypothetical protein
1.11	**BMAA2014**	hypothetical protein
1.06	**BMAA1384**	hypothetical protein
1.06	**BMA2875**	hypothetical protein
1.04	BMA0361	thioredoxin, authentic frameshift
1.01	**BMA0859**	hypothetical protein
-1.02	BMAA0427	TonB-dependent copper receptor
-1.04	BMA0665	phosphoadenosine phosphosulfate reductase, putative
-1.07	BMAA1196	transcriptional regulator, LysR family

Genes exhibiting ≥ 2-fold intensity (mRNA abundance) difference are listed. Loci in bold type are also differentially expressed in the human blood isolate (FMH) (see Table 4).

**Table 6:** Expression ratios of genes at or near sites of indels.

	**A. >2X UP REGULATED GENES NEAR INDELS**
**>2X Expression Altered Near Gene**	**Intergenic Indels**

BMAA1865	Human liver 2, BMAA1868
BMAA1865	Human blood 1, BMAA1866
BMAA1865	Human blood 5, BMAA1868
BMAA0112	FMH2, BMAA0117
	
	**B. EXPRESSION RATIOS OF INDEL GENES**

**Ratio**	**Intergenic Indels**

0.08	Human liver 1, BMA1135
0.29	Human liver 2, BMAA1868
0	Human liver 3, BMAA0375
0.47	Human blood 1, BMAA1866
-0.34	Human blood 2, BMAA0117
0.13	Human blood 3, BMAA0375
0.35	Human blood 4, BMAA1128
0.42	Human blood 5, BMAA1868
	
	**Indels within coding regions**

0.62	Human liver 1, BMAA0729
0.63	Human liver 2, BMA0328
0.04	Human liver 3, BMAA1903
0.06	Human liver 4, BMA0685
0.93	Human blood 1, BMA3028
-0.15	Human blood 2, BMAA0789
0.48	Human blood 3, BMA1903
